# Supplementary material for: Eating Behavior Associated with Food Intake in European Adolescents Participating in the HELENA Study
Source: Nutrients. 2022 Jul 24;14(15):3033. doi: 10.3390/nu14153033 (PMC9332602; doi:10.3390/nu14153033)
Supplement: Supplementary file 1 [file nutrients-14-03033-s001.zip › nutrients-1795850-supplementary.pdf]

SUPPLEMENTARY MATERIAL

File S1: Food groups combined based on their nutritional similarity.

Tables with results of Model 1= adjusted for the variables age, maternal education and BMI Z-score

Table S1. Mean (95% CI) intake of food groups by levels of the strength and motivation to eat component (EWI 1), in adolescents from the HELENA study.

**Food Groups**	**EWI 1 - Strength and motivation to eat**					
	**Boys**			**Girls**		
	Low<br>n = 781	High<br>n = 227	p	Low<br>n = 900	High<br>n = 286	p
Cereals and tubers (g/day)	325.8<br>318.5-333.2	354.4<br>340.7-368.2	**<0.001**	260.4<br>255.0-265.9	278.7<br>268.9-288.4	**0.001**
Sweets (g/day)	77.4<br>74.0-80.9	79.3<br>72.8-85.7	0.621	66.8<br>64.1-69.6	76.0<br>71.1-80.9	**0.002**
Dairy products (g/day)	324.2<br>307.8-340.5	328.2<br>297.7-358.7	0.820	251.1<br>240.2-262.0	247.7<br>228.3-267.1	0.767
Nuts, seeds, olives and avocado (g/day)	3.2<br>2.4-3.9	3.4<br>2.0-4.8	0.772	3.5<br>2.7-4.4	5.7<br>4.2-7.2	**0.015**
Alcoholic beverages (g/day)	24.5<br>15.9-33.2	39.9<br>23.8-55.9	0.101	5.7<br>2.5-9.0	5.3<br>-0.5-11.1	0.897
Chocolate (g/day)	26.8<br>24.5-29.2	34.5<br>30.1-38.9	**0.003**	20.0<br>18.5-21.5	25.0<br>22.3-27.7	**0.001**
Savory snacks (g/day)	9.2<br>7.9-10.4	12.7<br>10.3-15.0	**0.010**	5.7<br>6.5-9.8	8.4<br>7.0-9.8	**0.001**
Vegetable oils (g/day)	7.5<br>6.7-8.3	7.8<br>6.3-9.2	0.770	6.1<br>5.5-6.7	6.1<br>5.0-7.1	0.940
Margarine and lipids of mixed origins (g/day)	4.1<br>3.4-4.8	4.1<br>2.8-5.4	0.971	2.8<br>2.4-3.2	2.2<br>1.4-2.9	0.141
Butter and animal fats (g/day)	6.7<br>5.7-7.6	7.5<br>5.8-9.2	0.380	4.9<br>4.3-5.5	6.0<br>4.9-7.1	0.088
Sauces (g/day)	35.9<br>34.0-37.8	39.8<br>36.2-43.4	0.061	28.9<br>27.4-30.3	34.1<br>31.6-36.7	**0.001**

Pulses (g/day)	9.8<br>7.5-12.1	12.6<br>8.4-16.9	0.250	7.9<br>6.2-9.6	11.2<br>8.1-14.2	0.069
Vegetables (excl. potatoes) (g/day)	89.3<br>85.1-93.5	95.0<br>87.2-102.8	0.204	91.3<br>87.7-95.0	95.7<br>89.2-102.2	0.251
Fruits (g/day)	124.5<br>117.1-131.8	129.6<br>115.9-143.3	0.518	129.4<br>123.4-135.4	131.1<br>120.4-141-8	0.788
Soups, bouillon (g/day)	39.9<br>35.0-44.9	37.2<br>28.0-46.4	0.606	37.5<br>33.5-41.5	45.4<br>38.3-52.5	0.057
Water (g/day)	742.0<br>704.1-779.9	762.7<br>692.1-833.3	0.613	736.3<br>704.2-768.3	764.7<br>707.5-821.9	0.396
Cofee, tea (g/day)	43.3<br>36.1-50.6	46.0<br>32.5-59.5	0.734	50.8<br>43.8-57.8	51.7<br>39.2-64.2	0.904
Fruit and vegetables juices (g/day)	169.9<br>158.2-181.6	157.5<br>135.7-179.3	0.326	140.4<br>131.7-149.0	134.7<br>119.3-150.1	0.530
Sugar sweetened bevarages (g/day)	358.8<br>334.2-383.4	427.2<br>381.4-473.0	**0.010**	211.8<br>197.4-226.3	217.2<br>191.5-243.0	0.721
Meat and meat products (g/day)	163.7<br>157.9-169.5	162.0<br>151.2-172.8	0.789	127.3<br>123.1-131.6	139.0<br>131.5-146.5	**0.008**
Fish and fish products products (g/day)	20.4<br>18.8-22.0	20.3<br>17.3-23.3	0.950	19.9<br>18.5-21.4	19.7<br>17.1-22.3	0.875
Eggs (g/day)	13.5<br>12.4-14.7	12.4<br>10.3-14.5	0.340	11.2<br>10.3-12.1	10.7<br>9.1-12.3	0.613

CI= Confidence Intervals; EWI= The Eating Attitudes and Weight problems Inventory; HELENA = Healthy Lifestyle in Europe by Nutrition in Adolescence; g= grams; Significant difference in the ANCOVA(p<0.05).

Table S2. Mean (95% CI) intake of food groups by levels of the importance and impact of eating component (EWI 2), in adolescents from the HELENA study.

**Food Groups**	**EWI 2 - Importance and impact of eating**					
	**Boys**			**Girls**		
	Low<br>n= 770	High<br>n= 238	p	Low<br>n= 904	High<br>n= 282	p
Cereals and tubers (g/day)	332.2<br>324.8-339.7	332.5<br>319.9-345.9	0.977	262.6<br>257.1-268.1	272.1<br>262.3-281.9	0.098
	75.7	84.9	**0.012**	67.2	75.0	**0.007**

Sweets (g/day)	72.2-79.1	78.6-91.1		64.4-70.0	70.0-80.0	
Dairy products (g/day)	320.6<br>304.1-337.1	339.5<br>309.9-369.1	0.274	249.7<br>238.8-260.6	252.1<br>232.5-271.6	0.836
Nuts, seeds, olives and avocado (g/day)	2.8<br>2.1-3.6	4.4<br>3.0-5.8	0.052	3.7<br>2.9-4.6	5.2<br>3.6-6.7	0.105
Alcoholic beverages (g/day)	30.4<br>21.7-39.0	20.3<br>4.7-35.9	0.270	6.5<br>3.2-9.8	2.8<br>-2.9-8.87	0.284
Chocolate (g/day)	28.3<br>25.9-30.7	29.4<br>25.1-33.8	0.644	20.6<br>19.1-22.1	23.1<br>20.4-25.8	0.112
Savory snacks (g/day)	9.2<br>7.9-10.3	12.7<br>10.3-15.0	**0.010**	5.8<br>5.0-6.6	8.0<br>6.6-9.4	**0.008**
Vegetable oils (g/day)	7.2<br>6.4-8.0	8.9<br>7.4-10.3	**0.044**	5.7<br>5.1-6.2	7.6<br>6.5-8.6	**0.002**
Margarine and lipids of mixed origins (g/day)	4.4<br>3.7-5.1	3.1<br>1.8-4.4	0.070	2.9<br>2.5-3.3	1.8<br>1.0-2.5	**0.009**
Butter and animal fats (g/day)	7.3<br>6.3-8.2	5.5<br>3.9-7.2	0.072	5.2<br>4.6-5.8	5.2<br>4.1-6.3	0.931
Sauces (g/day)	36.0<br>34.1-38.0	39.3<br>35.7-42.8	0.113	30.0<br>28.5-31.4	30.8<br>28.1-33.4	0.608
Pulses (g/day)	9.6<br>7.3-11.9	12.9<br>8.8-17.0	0.178	8.3<br>6.6-10.0	10.0<br>7.0-13.1	0.330
Vegetables (excl. potatoes) (g/day)	88.0<br>83.8-92.1	99.1<br>91.5-106.7	**0.012**	90.4<br>86.8-94.0	98.8<br>92.2-105.3	**0.028**
Fruits (g/day)	121.5<br>113.9-128.6	139.9<br>126.7-153.1	**0.016**	130.8<br>124.8-136.7	126.7<br>115.9-137.4	0.516
Soups, bouillon (g/day)	38.4<br>33.4-43.3	42.4<br>33.5-51.3	0.440	39.0<br>35.0-42.9	40.9<br>33.7-48.0	0.645
Water (g/day)	716.6<br>678.8-754.6	843.8<br>775.5-912.0	**0.001**	743.9<br>711.9-775.9	740.5<br>683.0-798.0	0.919
Cofee, tea (g/day)	45.2<br>37.9-52.4	39.9<br>26.8-53.0	0.489	52.5<br>45.5-59.5	46.2<br>33.6-58.8	0.392
Fruit and vegetables juices (g/day)	173.3<br>161.6-185.1	147.0<br>125.9-168.2	**0.033**	140.9<br>132.3-149.5	133.0<br>117.5-148.4	0.382
Sugar sweetened bevarages (g/day)	376.9	365.4	0.659	220.0	191.1	0.055

	352.1-401.7	320.8-410.1		205.6-234.4	165.2-216.9	
Meat and meat products (g/day)	161.0<br>155.2-166.9	170.6<br>160.1-181.1	0.120	129.9<br>125.7-134.1	130.9<br>123.4-138.5	0.818
Fish and fish products products (g/day)	18.8<br>17.2-20.4	25.5<br>22.7-28.4	**<0.001**	19.9<br>18.5-21.4	19.7<br>17.1-22.3	0.899
Eggs (g/day)	12.6<br>11.5-13.7	15.5<br>13.4-17.5	**0.017**	10.5<br>9.6-11.4	12.8<br>11.2-14.4	**0.015**

CI= Confidence Intervals; EWI= The Eating Attitudes and Weight problems Inventory; HELENA = Healthy Lifestyle in Europe by Nutrition in Adolescence; g= grams; Significant difference in the ANCOVA(p<0.05).

Table S3. Mean (95% CI) intake of food groups by levels of the eating as a means of coping with emotional stress component (EWI 3) in adolescents from the HELENA study.

**Food Groups**	**EWI 3 - Eating as a means of coping with emotional stress**					
	**Boys**			**Girls**		
	Low<br>n=833	High<br>n= 175	p	Low<br>n= 886	High<br>n= 194	p
Cereals and tubers (g/day)	328.9<br>321.7-336.1	348.4<br>332.6-364.2	**0.028**	258.7<br>253.2-264.2	284.1<br>272.2-295.9	**<0.001**
Sweets (g/day)	77.6<br>74.2-80.9	79.1<br>71.8-86.5	0.713	67.6<br>64.8-70.4	74.3<br>68.2-80.3	0.051
Dairy products (g/day)	320.6<br>304.1-337.1	339.5<br>309.9-369.1	0.274	247.6<br>236.6-258.7	255.3<br>231.5-279.1	0.568
Nuts, seeds, olives and avocado (g/day)	3.1<br>2.3-3.8	4.0<br>2.4-5.6	0.309	3.5<br>2.7-4.3	5.8<br>4.0-7.6	**0.021**
Alcoholic beverages (g/day)	26.2<br>17.8-34.5	36.7<br>18.3-55.1	0.308	5.3<br>1.9-8.8	9.6<br>2.1-17.0	0.313
Chocolate (g/day)	28.3<br>25.9-30.7	29.4<br>25.1-33.8	0.644	21.3<br>19.7-22.8	21.5<br>18.1-24.8	0.929
Savory snacks (g/day)	10.1<br>8.8-11.3	9.5<br>6.8-12.2	0.699	5.8<br>5.0-6.6	8.3<br>6.6-10.0	**0.010**
Vegetable oils (g/day)	7.4<br>6.7-8.2	8.3<br>6.6-10.0	0.372	5.8<br>5.2-6.4	7.4<br>6.1-8.7	**0.026**
Margarine and lipids of mixed origins (g/day)	4.1<br>3.4-4.8	4.1<br>2.6-5.5	0.932	2.9<br>2.4-3.3	2.2<br>1.3-3.2	0.223

Butter and animal fats (g/day)	6.7<br>5.8-7.6	7.5<br>5.5-9.4	0.495	4.9<br>4.3-5.6	6.2<br>4.9-7.5	0.099
Sauces (g/day)	36.1<br>34.2-38.0	40.0<br>35.9-44.1	0.091	29.1<br>27.7-30.6	31.4<br>28.3-34.5	0.203
Pulses (g/day)	9.3<br>7.1-11.5	15.6<br>10.7-20.4	**0.022**	8.1<br>6.4-9.7	10.1<br>6.5-13.6	0.323
Vegetables (excl. potatoes) (g/day)	89.4<br>85.4-93.5	96.2<br>87.3-105.1	0.175	89.8<br>86.0-93.5	107.4<br>99.3-115.4	**<0.001**
Fruits (g/day)	122.6<br>115.5-129.7	139.9<br>124.3-155.5	**0.049**	131.4<br>125.4-137.5	122.9<br>109.9-135.9	0.243
Soups, bouillon (g/day)	38.8<br>34.0-43.6	41.8<br>31.3-52.3	0.608	39.2<br>35.1-43.3	46.9<br>38.1-55.7	0.121
Water (g/day)	751.0<br>714.3-787.7	725.9<br>645.3-806.6	0.580	728.8<br>696.2-761.5	808.5<br>738.1-878.9	**0.045**
Cofee, tea (g/day)	43.7<br>36.7-50.7	45.1<br>29.7-60.5	0.865	49.4<br>42.4-56.4	54.9<br>39.9-70.0	0.514
Fruit and vegetables juices (g/day)	169.5<br>158.1-180.8	156.1<br>131.2-181.0	0.340	139.1<br>130.2-148.0	141.9<br>122.8-161.1	0.793
Sugar sweetened bevarages (g/day)	366.9<br>343.0-390.7	409.1<br>356.7-461.5	0.151	218.5<br>204.0-233.0	199.4<br>168.1-230.6	0.277
Meat and meat products (g/day)	161.4<br>155.8-167.1	172.2<br>159.8-184.5	0.123	126.7<br>122.5-130.9	141.0<br>132.0-150.0	0.005
Fish and fish products products (g/day)	20.2<br>18.6-21.7	21.5<br>18.1-24.8	0.499	19.8<br>18.3-21.3	19.1<br>15.9-22.3	0.700
Eggs (g/day)	13.1<br>12.0-14.2	14.1<br>11.7-16.5	0.447	10.6<br>9.7-11.5	12.1<br>10.2-14.0	0.169

CI= Confidence Intervals; EWI= The Eating Attitudes and Weight problems Inventory; HELENA = Healthy Lifestyle in Europe by Nutrition in Adolescence; g= grams; Significant difference in the ANCOVA(p<0.05)
